# Rheological Study on the Thermoreversible Gelation of Stereo-Controlled Poly(*N*-Isopropylacrylamide) in an Imidazolium Ionic Liquid

**DOI:** 10.3390/polym11050783

**Published:** 2019-05-02

**Authors:** Zhi-Chao Yan, Chandra Sekhar Biswas, Florian J. Stadler

**Affiliations:** College of Materials Science and Engineering, Shenzhen Key Laboratory of Polymer Science and Technology, Guangdong Research Center for Interfacial Engineering of Functional Materials, Nanshan District Key Lab for Biopolymers and Safety Evaluation, Shenzhen University, Shenzhen 518055, China; chandra01234@gmail.com (C.S.B.); fjstadler@szu.edu.cn (F.J.S.)

**Keywords:** Poly(*N*-isopropylacrylamide), tacticity, ionic liquid, rheology

## Abstract

The thermoreversible sol-gel transition for an ionic liquid (IL) solution of isotactic-rich poly (*N*-isopropylacrylamides) (PNIPAMs) is investigated by rheological technique. The meso-diad content of PNIPAMs ranges between 47% and 79%, and molecular weight (*M*_n_) is ~35,000 and ~70,000 g/mol for two series of samples. PNIPAMs are soluble in 1-butyl-3-methylimidazolium bis(trifluoromethanesulfonyl) imide ([BMIM][TFSI]) at high temperatures but undergo a gelation with decreasing temperatures. The transition temperature determined from *G*’-*G*” crossover increases with isotacticity, consistent with the previous cloud-point result at the same scanning rate, indicating imide groups along the same side of backbones are prone to be aggregated for formation of a gel. The transition point based on Winter-Chambon criterion is on average higher than that of the *G*’-*G*” crossover method and is insensitive to tacticity and molecular weight, since it correlates with percolation of globules rather than the further formation of elastic network (*G*’ > *G*”). For the first time, the phase diagram composed of both *G*’-*G*” crossover points for gelation and cloud points is established in PNIPAM/IL mixtures. For low-*M*_n_ PNIPAMs, the crossover-point line intersects the cloud-point line. Hence, from solution to opaque gel, the sample will experience two different transitional phases, either clear gel or opaque sol. A clear gel is formed due to partial phase separation of isotactic segments that could act as junctions of network. However, when the partial phase separation is not faster than the formation of globules, an opaque sol will be formed. For high-*M*_n_ PNIPAMs, crossover points are below cloud points at all concentrations, so their gelation only follows the opaque sol route. Such phase diagram is attributed to the poorer solubility of high-*M*_n_ polymers for entropic reasons. The phase diagram composed of Winter-Chambon melting points, crossover points for melting, and clear points is similar with the gelation phase diagram, confirming the mechanism above.

## 1. Introduction

Stimuli-responsive gels have received great attention owing to their scientific interest and potential applications [1,2]. The merit of these materials lies on their rapid and reversible response to environmental changes, such as temperature, pH, light, and stress. For thermosensitive gels, the response relies on the abrupt change in solubility upon cooling or heating. As a typical thermo-responsive polymer, poly (*N*-isopropylacrylamide) (PNIPAM) in aqueous solutions exhibits a lower critical solution temperature (LCST) phase behavior around ambient temperatures (≈33 °C) [3]. These notable features make PNIPAMs widely used in thermoreversible hydrogel for sensing and biomedical materials [4,5,6,7]. Despite the appealing applications, however, PNIPAM hydrogels suffer from the volatility of water, preventing their use at high temperatures and storage at open atmosphere. Moreover, as the major component, water cannot offer additional functionality to the gel besides polymers. In order to solve the above problems, non-volatile and functional solvents are desired to substitute water. Ionic liquids (ILs) are non-volatile room-temperature molten salts with thermal and chemical stability and conductivity [8]. Owing to these properties, ILs are ideal solvents for making conductive gels, which could be used at open atmosphere over a wide range of temperatures [9,10,11,12]. The stimuli-responsive properties can be readily tailored by changing the chemical structures of the ions or by mixing with different solvents, without modification of polymer structures [13,14]. Such convenience of manipulating gels for a target purpose generates interest in developing polymeric materials in ILs, especially the thermoreversible ionogels [15,16,17,18,19,20].

The ionic liquid, 1-alkyl-3-methylimidazolium bis(trifluoromethanesulfonyl)imide ([XMIM][TFSI], X = E for ethyl, and X = B for butyl), has proven to be a solvent for PNIPAMs [21]. The PNIPAM/IL mixture exhibits an upper critical solution temperature (UCST) phase behavior [21,22,23,24], opposite to aqueous solutions [3,25]. Based on this property, PNIPAM and its copolymers have been employed to make thermoresponsive ionogels in ILs by self-assembly. He et al. [26] synthesized poly (*N*-isopropyl acrylamide-*b*-ethylene oxide-*b*-*N*-isopropyl acrylamide) (PNIPAM-*b*-PEO-*b*-PNIPAM) triblock copolymers and blended them with [EMIM][TFSI]. Above the UCST-type transition temperature, PNIPAM blocks are expanded coils, while below the transition temperature, PNIPAM blocks experience coil-to-globule transition and become aggregates, which are connected by PEO coils, finally forming a gel. Ueki et al. [16] produced poly (benzyl methacrylate-*b*-*N*-isopropyl acrylamide) (PBnMA-*b*-PNIPAM) diblock copolymers, with PBnMA and PNIPAM blocks exhibiting LCST and UCST phase separations in ILs, respectively. As a result, a doubly thermosensitive self-assembly was observed in [EMIM][TFSI], where a homogeneous solution only exists in a window between UCST and LCST phase boundary. Likewise, Lee et al. [27] reported a doubly thermoresponsive diblock copolymer, poly (ethylene oxide-*b*-*N*-isopropylacrylamide) (PEO-*b*-PNIPAM), that exhibits both an upper critical micellization temperature (UCMT) and a lower critical micellization temperature (LCMT) in 1-ethyl-3-methylimidazolium tetrafluoroborate ([EMIM][BF_4_]), 1-butyl-3-methylimidazolium tetrafluoroborate ([BMIM][BF_4_]), and their blends. By varying the mixing ratio of two ILs, both UCMT and LCMT can be readily tuned in a mixed solvent. Ueki et al. [28] further developed a triblock ABA copolymer, denoted as P(AzoMA-*r*-NIPAM)-*b*-PEO-*b*-P(AzoMA-r-NIPAM), with AzoMA being 4-phenylazophenyl methacrylate, which is sensitive to photo-stimulus. By combining the photosensitive (AzoMA) and thermosensitive (NIPAm) blocks, a doubly reversible gel in response to either photo-and thermo-stimuli, was obtained in the IL 1-butyl-3-methylimidazolium hexafluorophosphate ([BMIM][PF_6_]).

To clarify the mechanism of thermoresponse in PNIPAM/IL mixtures and guide the design of ionogels, fundamental researches have been performed to investigate the effect of molecular weight, concentration, and structure on their phase behavior. For example, Asai et al. [22] reported the cloud point of PNIPAM/[EMIM][TFSI] increases with molecular weight (*M*_n_) and polymer concentration by dynamics light scattering. Using small-angle neutron scattering, they found the chain size at dilute regime decreases, while the correlation length at semidilute regime increases as approaching UCST. The Flory−Huggins interaction parameter, *χ*, becomes larger with cooling, and exceeds 0.5 around 45 °C. Besides, molecularly dispersed PNIPAM chains are found to still remain in ILs even after macroscopic phase separation, distinct from a complete phase separation in a molecular level in aqueous solutions. De Santis et al. [23] confirmed the abovementioned *M*_n_ dependence and firstly reported the increase in phase transition temperature with isotacticity, although the meso-diad content (denoted as *m*) in their PNIPAMs only ranges a narrow variation from 55% to 66% and the concentration is fixed at 1%. Wang et al. [29] experimentally investigated the phase separation mechanism in PNIPAM/[EMIM][TFSI] solutions by infrared spectroscopy and attributed it to the desolvation of PNIPAMs. Ueki et al. [30] reported the isomeric effect on the UCST-type separation of random copolymers comprising NIPAM and AzoMA in [EMIM][TFSI] and confirmed that trans-isomer leads to an increase in the phase separation temperature, whereas the cis-isomer contributes to a decrease in the phase separation temperature. So et al. [31] showed how the incorporation of comonomers with different hydrogen-bonding capacities into PNIPAM affects the UCST behavior and the corresponding sequential and reversible self-folding. Very recently, we have studied the phase diagram of two series (depending on molecular weight) of PNIPAMs with varying isotacticity in [BMIM][TFSI] by turbidity measurement using a concentration range of 1–12.5% (w/v) [24]. It was observed that the UCST-type phase separation temperature increases with isotacticity and molecular weight. The phase diagram was firstly determined for a highly isotactic PNIPAM (*m* ≈ 78%), where a clear UCST peak was observed at low concentrations, similar with the conventional polymer solution, implying an entropy-driven dissolution mechanism.

In spite of the abovementioned research on the coil-globule transition, however, the sol-gel transition in PNIPAM/IL mixtures has not been systematically studied to the best of our knowledge. Heretofore, the study on sol-gel transition of PNIPAMs mainly exists in aqueous and organic solutions. Nakano et al. [32] studied the thermoreversible gelation of isotactic-rich poly (*N*-isopropylacrylamide) in water and observed the gelation happens below the LCST cloud-point temperatures. Upon heating, solutions undergo transparent gels first before they become turbid. Tanaka et al. [33] attributed this phase diagram to the preferential dehydration of meso-diad segments on the chain, whose partial phase separation creates physical crosslinkers for a transparent gel. Upon further heating, other segments are also dehydrated, so that the whole chain transfers to globules and the system becomes turbid. Such partial phase separation was also reported in isotactic-rich PNIPAM/benzyl alcohol blends [34], where a transparent gel was formed below the UCST-type transition temperatures, and the transition temperature increases with the meso-diad content. As molten salts, ILs have different interactions with PNIPAM in comparison to water and organic solvents. The structure-property relationship in aqueous and organic gel does not necessarily apply for the IL gels. Therefore, the gelation behavior in PNIPAM/IL is highly desired to be investigated for both scientific interest and application-based importance in guiding the design of smart ionogels. To this end, the effect from several key factors, including tacticity, molecular weight, and concentration, on the sol-gel transition need to be clarified. The phase diagram from gelation should be established and compared with that from cloud-points.

In this work, we for the first time studied the reversible gelation in isotactic-rich PNIPAM/IL blends using rheological technique. PNIPAMs with a wide range of tacticity (*m* = 47–79%) and different molecular weights were employed to mix with [BMIM][TFSI]. The dependence of sol-gel transition on tacticity, concentration, and molecular weight was examined and compared with the coil-globule transition determined from cloud points. A complete phase diagram was established accordingly. This research will provide a guideline for the design of thermoresponsive ionogels based on PNIPAMs.

## 2. Experiments

### 2.1. Sample Information

The synthesis and characterization of stereo-controlled PNIPAMs was described in Reference [34]. Table 1 lists the number-average molecular weight (*M*_n_), polydispersity (PDI), and tacticity of the investigated PNIPAMs. The tacticity is defined as the percentage of the meso-diad content, which was determined by ^1^H NMR [34]. Based on molecular weight, PNIPAMs are denoted as *m* and *Hm* series. For *m*-series, *M*_n_ is from 34,900 to 42,600 g/mol with PDI in between 1.22 and 1.26. Their tacticities are 47%, 58%, 66%, and 79%, respectively. For *Hm*-series, *M*_n_ is doubled and ranging from 60,200 to 85,700 g/mol with PDI in between 1.26 and 1.49. Their tacticities are 48%, 57%, 67%, and 78%, respectively.

**Scheme 1:**
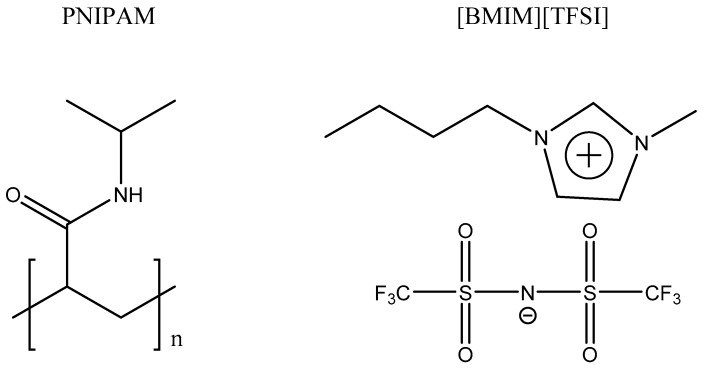
Structure of PNIPAM and [BMIM][TFSI].

The ionic liquid was 1-butyl-3-methylimidazolium bis(trifluoromethanesulfonyl)imide ([BMIM][TFSI]), from the Center for Green Chemistry and Catalysis of Lanzhou Institute of Chemical Physics, Chinese Academy of Sciences. [BMIM][TFSI] was dried at 80 ℃ under vacuum to remove residue water before use. The polymer/IL solution with a particular concentration (w/v %) was prepared with the help of a cosolvent (tetrahydrofuran). After sufficient evaporation in a fume-hood, mixtures were dried in a vacuum to remove residue cosolvent and bubbles. Details for mixture preparation was reported in Reference [24]. The structures of PNIPAM and [BMIM][TFSI] are presented in Scheme 1.

### 2.2. Rheological Measurements

The solutions were characterized rheologically using a cone-plate geometry with cone angle of 2° and diameter of 25 mm on an Anton Paar 302 rheometer. Temperatures were controlled by Peltier plate. Samples were subjected to temperature ramps between 10 and 60 ℃ at a heating and cooling rate of 1 °C/min with an angular frequency of 10 rad/s. Heating was performed first followed by a cooling procedure. The strain amplitude is kept at 0.1%, within the linear viscoelastic regime at all temperatures. Dynamic frequency sweep was also performed on each sample from 10 to 40 ℃ with proper strain to ensure the linear viscoelasticity and enough waiting time to allow the material to equilibrate.

## 3. Result and Discussion

### 3.1. Tacticity Dependence

**Figure 1:**
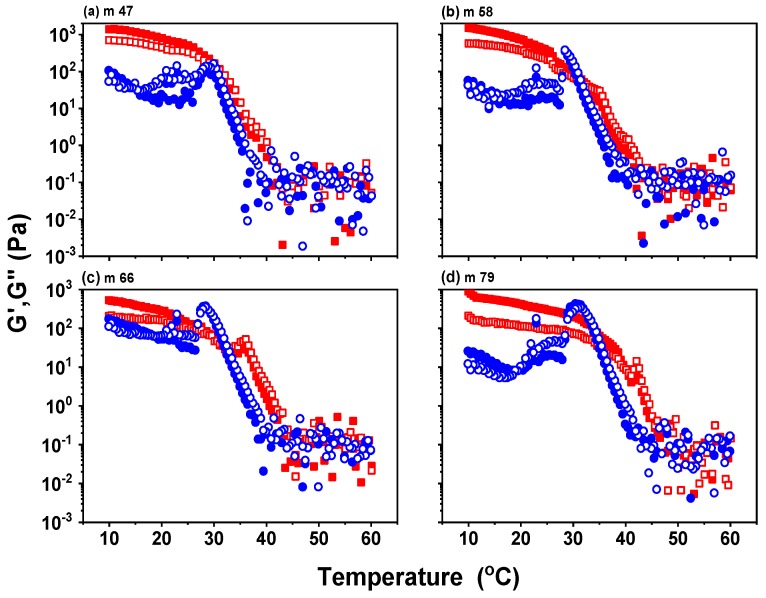
Variations in storage (*G*’, filled symbol) and loss (*G*”, open symbol) moduli of 5% (**a**) m47, (**b**) m58, (**c**) m66, and (**d**) m79 solutions as a function of temperature at a frequency of *ω* = 10 rad/s and a strain amplitude of *γ* = 0.1%. Symbols (■, □) and (●, ○) are for heating and cooling sweeps, respectively.

The temperature dependence of storage (*G*’) and loss (*G*”) moduli for 5% m series in ILs is shown in Figure 1. In heating procedure, moduli first exhibit a moderate decrease at the regime where *G*’ > *G*”. Then, an abrupt decrease in moduli occurs with *G*” gradually becoming larger than *G*’, reflecting a gel-to-sol transition. At higher temperatures (in solution state), data are scattered because the phase angle in oscillatory measurement is close to 90°, i.e., close to Newtonian, which makes *G*’ difficult to be precisely determined. Furthermore, the magnitude of the complex modulus |G*| is below 1 Pa, which corresponds to a torque of 1 nN∙m for the setup and parameters used, close to the resolution limit of the rheometer. In the cooling procedure, the sample state changes from solution to gel, proving the reversibility of the transition. An increase in moduli is observed followed by the recovery that *G*’ becomes larger than *G*”. The drop of moduli once the gel state is obtained is due to the slippage of sample, considering the sample becomes heterogeneous and solvophobic, which leads to a phase separation, partially at the interface between geometry [35]. Despite this artefact, the crossover of *G*’ and *G*”, which corresponds to the gelation transition, appears before this drop, so the final result is not affected. The gel-to-sol transition is located at a higher temperature than the sol-to-gel transition for all samples. This hysteresis is consistent with that in transmittance measurement [24], where the globule-to-coil transition is believed to be retarded by the extra hydrogen bonding among polymers in the aggregates with respect to the coil-to-globule transition [36,37]. Such hydrogen bonding mechanism may also account for the hysteresis in the sol-gel transition here, since the extra hydrogen bonding can kinetically delay the breaking of clusters that are the crosslinker of the gel network.

**Figure 2:**
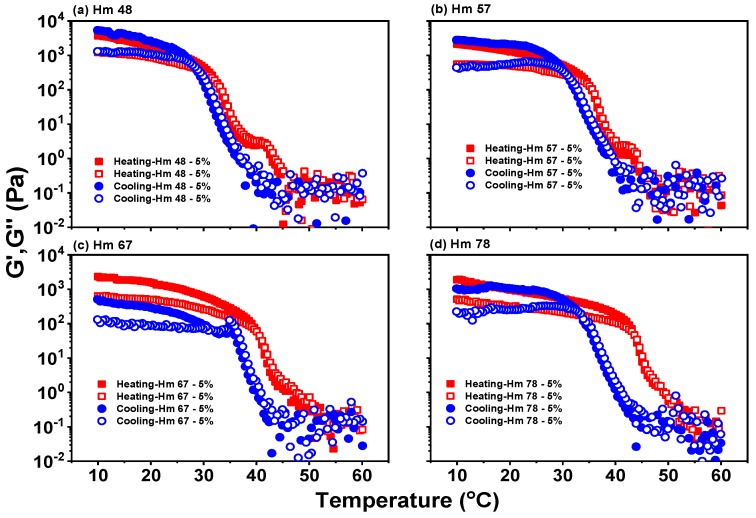
Variations in storage (*G*’, filled symbol) and loss (*G*”, open symbol) moduli of 5% (**a**) Hm48, (**b**) Hm58, (**c**) Hm67, and (**d**) Hm78 solutions as a function of temperature at a frequency of *ω* = 10 rad/s and a strain amplitude of *γ* = 0.1%. Symbols (■, □) and (●, ○) are for heating and cooling sweeps, respectively.

For Hm series, similar heating/cooling evolutions in rheological behavior are observed in Figure 2. Compared with m-series, the sol-gel transition in Hm appears at higher temperature, as the case in transmittance measurement [22,23,24,38]. This is attributed to a strong entropic contribution to the phase separation for high-*M*_n_ polymers in the thermodynamic aspect [24,39], which favors the forming of clusters as crosslinkers. The hysteresis in Hm also increases with isotacticity, and the amplitude is similar with that in m series, implying its insensitivity to molecular weight. In previous reports [23,24], the hysteresis in transmittance is not *M*_n_ dependent above 44,000 g/mol, because chain associations within aggregates are similar for different *M*_n_. This could be used to account for the gelation hysteresis and implies the forming/breaking of aggregates (crosslinkers of gels) governs the gelation/melting procedure.

**Figure 3:**
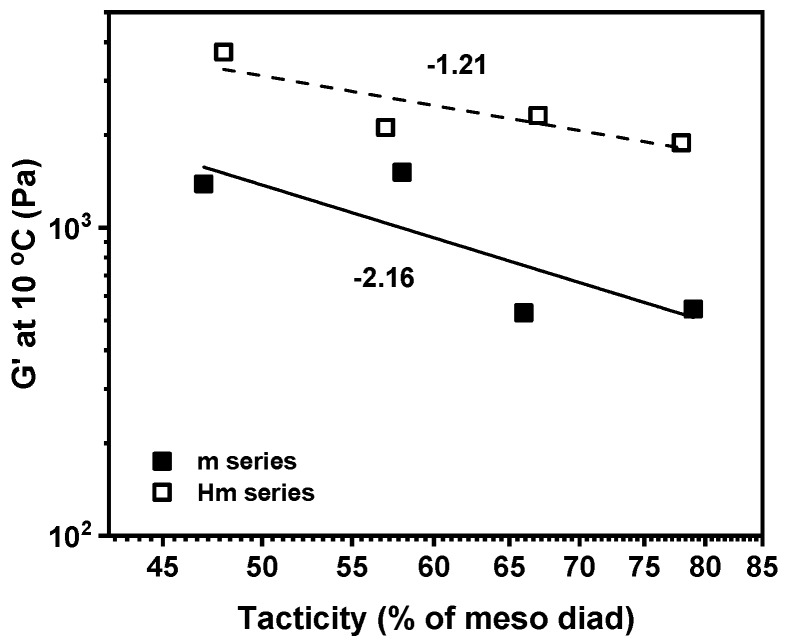
Comparison between *G*’ of m and Hm series at 10°C (heating scan) as function of tacticity.

To assess the gel network of different tacticity, we compared their storage moduli at 10 °C on heating curves in Figure 3, which are proportional to the density of network. For both m and Hm series, *G*’ slightly decreases with isotacticity, showing a scaling power law slope −2.2 and −1.2, respectively. This dependence is opposite to the case of PNIPAMs in benzyl alcohol [34], where the plateau *G*’ increases with isotacticity. However, we note that PNIPAM/benzyl alcohol gel is transparent, while the PNIPAM/IL gel is opaque. In a transparent gel, partial phase separation takes place only in solvophobic isotactic segments. These desolvated segments act as junctions to connect other segments that are still dissolved. With increasing content of meso-diad on the chain, the number of junctions increases, and hence the gel network is better linked. In an opaque gel, however, macroscopic phase separation takes places to a much larger extent. Segments are mainly aggregated into globules rather than left in ILs to act as a bridging chain. This situation is intensified with increasing isotacticity, reducing the number of connections among globules, and thus thinning out the network. The storage moduli of Hm are higher than m series, reflecting a better-connected gel because long chains have a higher probability to bridge different globules and increase the number of interglobular association [34,40,41].

Two methods are employed to extract the phase transition temperatures from rheological data. First, we adopt the crossover of *G*’ and *G*” at dynamic temperature sweep, which has been widely used to define transition temperature in hydro- and iono-gels formed by polymers’ phase separation [34,42,43,44,45,46,47,48,49,50,51,52]. Such a method can reflect the scanning rate dependence, making its results comparable with cloud points, which were also measured at 1 °C/min [24]. However, we note that this method suffers the problem of frequency dependence, making it not a rigorous determination of transition point. Therefore, we only refer to this point as “crossover point” in the following discussion. Another method is based on Winter and Chambon criterion [53,54,55,56], where the transition point is determined rheologically by finding the temperature at which the storage and loss moduli share the same power-law dependence on frequency. This method is independent of test frequency and can reflect the equilibrium transition temperature, which is insensitive to scanning rate. Lodge and coworkers [44] compared these two methods by investigating the gelation of PNIPAM-*b*-PEO-*b*-PNIPAM triblock copolymers in [EMIM][TFSI], where polymers have a molecular weight of 29,000 g/mol and concentration of 10%, close to our systems. They found both methods generate similar transition temperatures, with the crossover method being 17 °C and the Winter-Chambon criterion being 20°C. They further observed the consistency of two methods in poly (ethylene-alt-propylene-*b*-ethylene oxide-*b*-*N*-isopropylacrylamide) aqueous solutions [46] and poly (phenylethyl methacrylate-*b*-methyl mechacrylate-*b*-phenylethyl methacrylate) IL solutions [45]. In the next sections, we use both methods to extract transition temperatures and compare their results.

**Figure 4:**
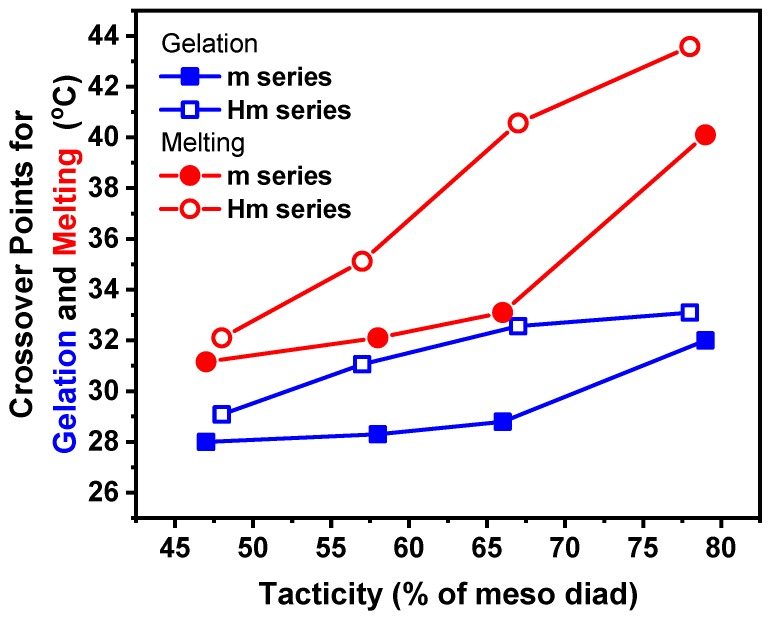
The crossover point for melting (●, ○) and gelation (■, □) procedures vs the tacticity for m (solid symbol) and Hm (open symbol) series.

The transition points corresponding to both sol-to-gel and gel-to-sol procedures are first determined by the crossover of *G*’ and *G*” and plotted in Figure 4 as a function of meso-diad content (tacticity). Both temperatures monotonically increase with isotacticity, consistent with the tendency of coil-globule transition [23,24], implying the structural factors for phase separation also apply to the gelation procedure. Ray et al. [25] proposed that highly isotactic PNIPAMs have more side groups aligned on the same side of backbones, which improves hydrogen bonding among amide groups and solvophobic association among isopropyl groups in the aggregates. Since aggregates act as the crosslinker within gels, the formation and melting of the gel is affected by tacticity. Likewise, the crossover point for melting PNIPAM/benzyl alcohol gels also increases with isotacticity [34]. In spite of the same tendency in both gels, however, the underlying mechanisms are different. The increase in crossover temperatures for melting PNIPAM/IL gels reflects the increasing difficulty to disaggregate globules, which are formed by the whole polymer chain, whereas the same dependence for PNIPAM/benzyl alcohol is attributed to the increasing number of small-size junctions in the network of transparent gels, which are induced by the partial phase separation of isotactic-rich segments on the chain. The crossover point for melting procedure is higher than that for gelation. With increasing isotacticity, their difference, Δ*T*_c_, is enlarged, which is inconsistent with the transmittance results where hysteresis is insensitive to tacticity [24]. The value of Δ*T*_c_ is in the same magnitude as in transmittance transition only when *m* is lower than 60%. For highly isotactic PNIPAMs (*m* > 65%), however, Δ*T*_c_ in sol-gel transition is significantly larger. This result implies the chain that associates clusters may introduce long-range interactions, which can improve the difficulty in both forming and breaking of clusters. Such a long-range effect is negligible at low isotacticity since the inter-and intra-chain interactions are relatively weak, but becomes important at high isotacticity where polymer interactions are strengthened. The transition temperatures of Hm series are higher than those of m series, which can be attributed to a stronger entropic contribution to the phase transition from high molecular weight [24]. The difference in transition temperatures between Hm and m series also increases with isotacticity, because the intrachain interactions in high-*M*_n_ polymers are relatively important and hence prone to be strengthened by the extra interaction from isotactic structures, with respect to low-*M*_n_ polymers.

**Figure 5:**
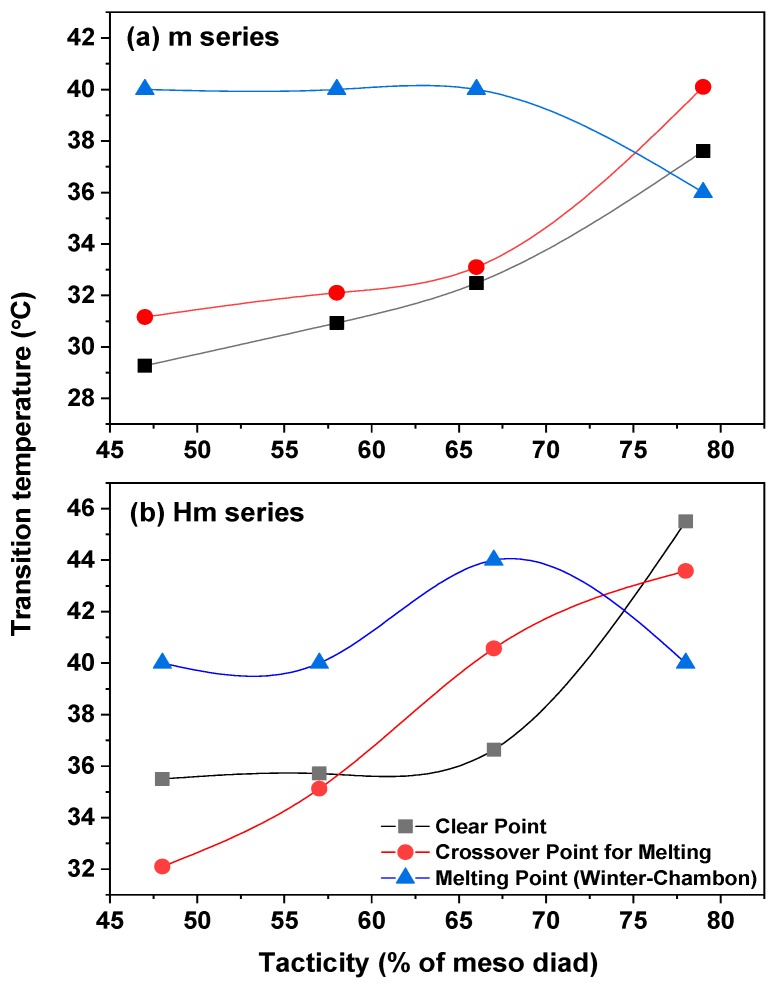
The *G*’-*G*” crossover point for melting procedure (●), the melting point determined by Winter-Chambon criterion (▲), and the clear point determined by transmittance measurement (■), as a function of the tacticity for (**a**) m and (**b**) Hm series with concentration of 5%.

Dynamic frequency sweep was performed at different temperatures by a heating route (Figures S1 and S2 for m and Hm series, respectively, shown in Supporting Information). The melting temperature was determined through Winter-Chambon criterion [53,54,55,56]. Since the sol-gel transition point is characterized by the identical power-law dependence on frequency for *G*’ and *G*”, the frequency-independent loss tangent tanδ (= *G”/G*’) has been extensively employed to determine the critical transition temperature, time, and concentration [57,58,59]. In this spirit, tanδ is plotted as a function of temperature at different frequencies (Figures S3 and S4 for m and Hm series, respectively, shown in Supporting Information), and the melting temperature was determined via an intersecting or closest point of tanδ curves [60]. Figure 5a illustrates the melting points from Winter-Chambon criterion, *G*’-*G*” crossover points for melting, as well as the clear point from transmittance measurement for m series [24]. The crossover point shares the same tacticity dependence as the clear point, considering both were measured at a 1 °C/min scanning rate. The transition point determined by Winter-Chambon criterion, however, is higher than the *G*’-*G*” crossover point at *m* = 47~66%, because the percolation of small-size globules from partial phase separation of segments (frequency-independent tanδ) takes place at higher temperature than the further development of the elastic network (*G*’ > *G*”). At *m* = 79%, the Winter-Chambon transition temperature is close to the *G*’-*G*” crossover value, implying the percolation of globules and the formation of the elastic gel happen simultaneously. Such synchronization could be attributed to the fact that high-*m* PNIPAMs favor the phase separation of the global chain rather than local segments upon percolation, which bypasses the grow-up of globule junctions and instantly forms an elastic gel. Figure 5b compared the abovementioned three kinds of temperatures for Hm series. Likewise, the crossover points monotonically increase with isotacticity as clear points. The Winter-Chambon melting points are higher than crossover values except for *m* = 78%, consistent with the m series and can be explained in the same spirit. The average value of all Winter-Chambon melting points in both m and Hm series in Figure 5 is 40 ± 2.2 °C, indicating the percolation temperature is lack of obvious tacticity and molecular weight dependence, different from the monotonic increase in the *G*’-*G*” crossover point, which reflects the formation of elasticity.

### 3.2. Concentration Dependence

**Figure 6:**
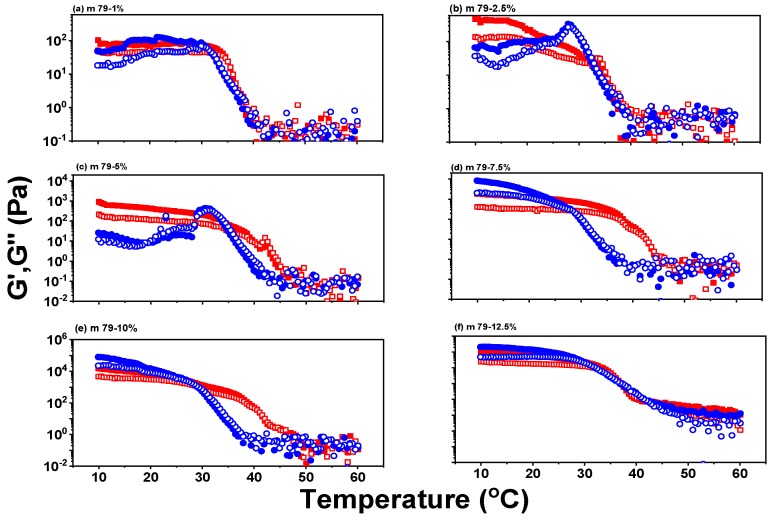
Variations in storage (*G*’, filled symbol) and loss (*G*”, open symbol) moduli for m79 solutions at concentrations of (**a**) 1%, (**b**) 2.5%, (**c**) 5%, (**d**) 7.5%, (**e**) 10%, and (**f**) 12.5% as a function of temperature at a frequency of *ω* = 10 rad/s and a strain amplitude of *γ* = 0.1%. Symbols (■, □) and (●, ○) are for heating and cooling sweeps, respectively.

The temperature dependence of moduli for m79 at different concentrations is shown in Figure 6. The reversible gelation procedure is observed at all concentrations. At 7.5~12.5%, the recovered moduli are even higher than those before melting. This is probably because the recovered gel bears a better-established network than the original gel, which experienced both long-term storage and structural disturbance during sample loading. The heating transition happens at higher temperatures than cooling transition, with their difference increasing first and then decreasing, reaching the maximum at the intermediate concentration (7.5%). Such concentration dependence of hysteresis is different from the concentration-independent result in the transmittance measurements [24]. The larger hysteresis at intermediate concentrations can be attributed to the strengthened long-range interactions due to the higher possibility of inter-cluster association. However, the increase in the inter-cluster contact is at the cost of intra-cluster association, so at the highest concentrations, the hysteresis is reduced since the inside of clusters is not as strongly bonded as at low concentrations and easier to be disaggregated during heating.

**Figure 7:**
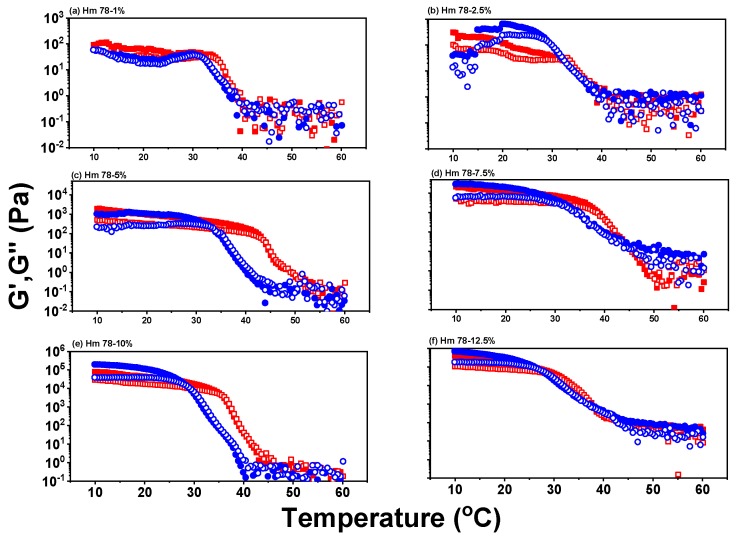
Variations in storage (*G*’, filled symbol) and loss (*G*”, open symbol) moduli for Hm78 solutions at concentrations of (**a**) 1%, (**b**) 2.5%, (**c**) 5%, (d) 7.5%, (**e**) 10%, and (**f**) 12.5% as a function of temperature at a frequency of *ω* = 10 rad/s and a strain amplitude of *γ* = 0.1%. Symbols (■, □) and (●, ○) are for heating and cooling sweeps, respectively.

The temperature dependence of moduli for Hm78 at different concentrations is shown in Figure 7. Since Hm series have the higher molecular weight, their moduli at gel regime are higher and the transition regime locates at higher temperature in comparison to m series for entropic reason. Like m series, the gel-state moduli of Hm monotonically increase with concentration, and the hysteresis between heating and cooling exhibits a maximum at the intermediate concentration. Rheological data of Hm are less scattered owing to the high stiffness of the gel. Besides, the recovered moduli are higher than the original ones at high concentrations, consistent with the results in m series, confirming a denser mesh in reestablished networks.

**Figure 8:**
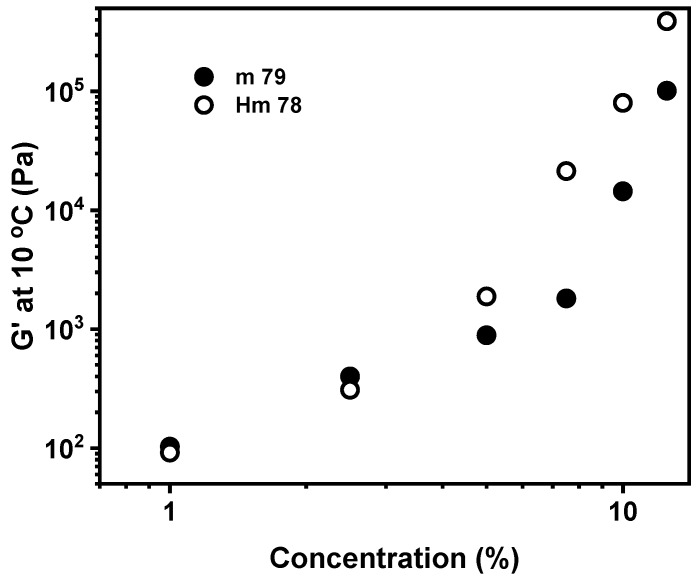
Comparison between *G*’ of m79 and Hm78 at 10 °C as a function of concentration.

The concentration dependence of *G*’ at 10°C on heating curves is shown in Figure 8. With increasing polymer concentrations, the moduli at gel regime gradually increase because the network mesh becomes denser. Unlike entangled polymer solutions [39], there is not a scaling-law type relationship between moduli and concentration. Instead, moduli increase moderately at low concentrations and rise rapidly at higher concentrations. Interestingly, moduli of m79 and Hm78 are similar at low concentration, suggesting that isolated globules, rather than associating chains, are preferentially formed when polymers are scarce, which makes the gel network too loose to be affected by molecular weight. Until concentration is high enough, the association among globules could be established and the gel becomes increasingly strong. This phenomenon is different from that of PNIPAMs in benzyl alcohol, where moduli increase rapidly at low concentrations and then tend to saturate at high concentrations [34]. This is because the partial phase separation mainly generates small-size junctions rather than large globules. The number of junctions significantly increases at low concentrations but approaches saturation at high concentrations. The upturn of data occurs at a lower concentration for Hm78 than m79, because the high molecular weight of Hm78 allows for a lower network percolation concentration. Similar results were also observed in PNIPAM/benzyl alcohol [34], where the plateau *G*’ moduli for Hm78 appears at lower concentrations than that for m79, indicating a lower percolation concentration for Hm.

**Figure 9:**
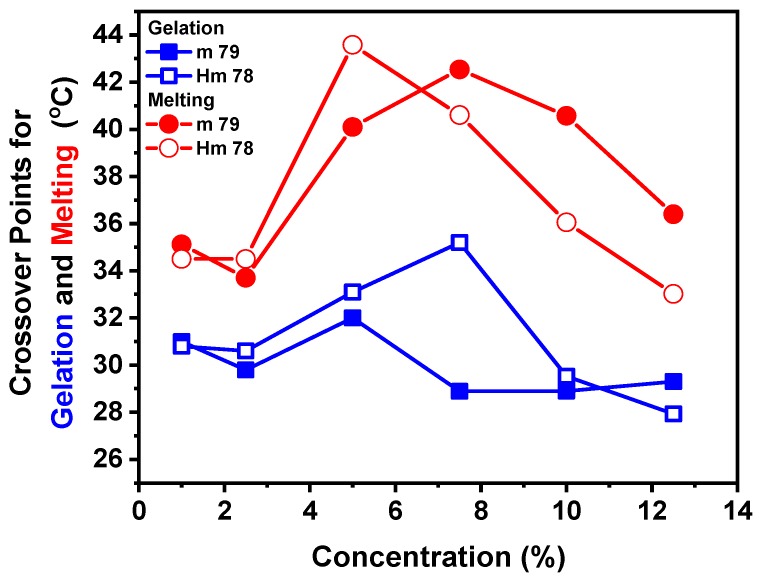
The crossover point for melting (●, ○) and gelation (■, □) procedures vs the concentration for m79 (solid symbol) and Hm78 (open symbol) solutions.

The crossover points for melting and gelation procedure are plotted as function of concentration in Figure 9. The two kinds of temperatures exhibit a UCST-type phase diagram, consistent with our previous observation in the transmittance method [24]. This asymmetric UCST phase transition is similar with that of the conventional polymer solutions [39], so an entropy driven dissolution mechanism was expected to be responsible for this behavior. Additionally, the similarity between sol-gel and coil-globule transitions implies the aggregation and disaggregation of clusters govern the gelation and melting procedures, respectively. The crossover points for melting are higher than those for gelation because the disaggregation of clusters is relatively delayed due to extra interactions inside clusters [23,36,37]. Furthermore, the difficulties of both bridging and breaking the inter-cluster associations further enlarge the hysteresis. As for the molecular weight effect, the crossover points for gelation of m79 and Hm78 are approximately overlapped at low and high concentration limits, while at intermediate concentrations, crossover points of Hm are higher than m because of the entropically induced poor solubility for higher-*M*_n_ samples. On the other hand, the crossover points for melting of m79 and Hm78 are close at low concentration limit, but at higher concentrations, the peak of m79 locates at the right side of Hm78 samples, with the data at the highest three concentrations being even larger than those of Hm78. This result is in conflict with the cloud-point phase diagram where phase separation temperatures of Hm78 are always higher than those of m79 at a fixed concentration [24]. This anomalous phenomenon could be tentatively attributed to the significant hysteresis for m79 at high concentrations. At a given concentration, the number density of chains in solutions is higher for low-*M*_n_ PNIPAMs than high-*M*_n_ PNIPAMs. As a result, low-*M*_n_ PNIPAMs favors the establishment of the stable interchain-associated aggregates, while high-*M*_n_ PNIPAMs are relatively isolated so that they prefer to form the less stable intrachain-associated aggregates [23]. This molecular weight effect seems to be amplified in the sol-gel transition where the stability of interchain associations plays an important role in connecting clusters rather than just agglomerating chains into globules. At last, we note that in PNIPAM/benzyl alcohol [34], the crossover point for gelation monotonically increases with concentration and lacks an UCST peak. Such difference lies in the distinct constitution within two kinds of gels. While the gelation of PNIPAM/IL is governed by the coil-to-globule procedure, the gelation of PNIPAM/benzyl alcohol mainly depends on the number of junctions formed by partial phase separation of isotactic-rich segments, which monotonically increases with polymer concentration and results in a higher transition temperature at high concentrations.

**Figure 10:**
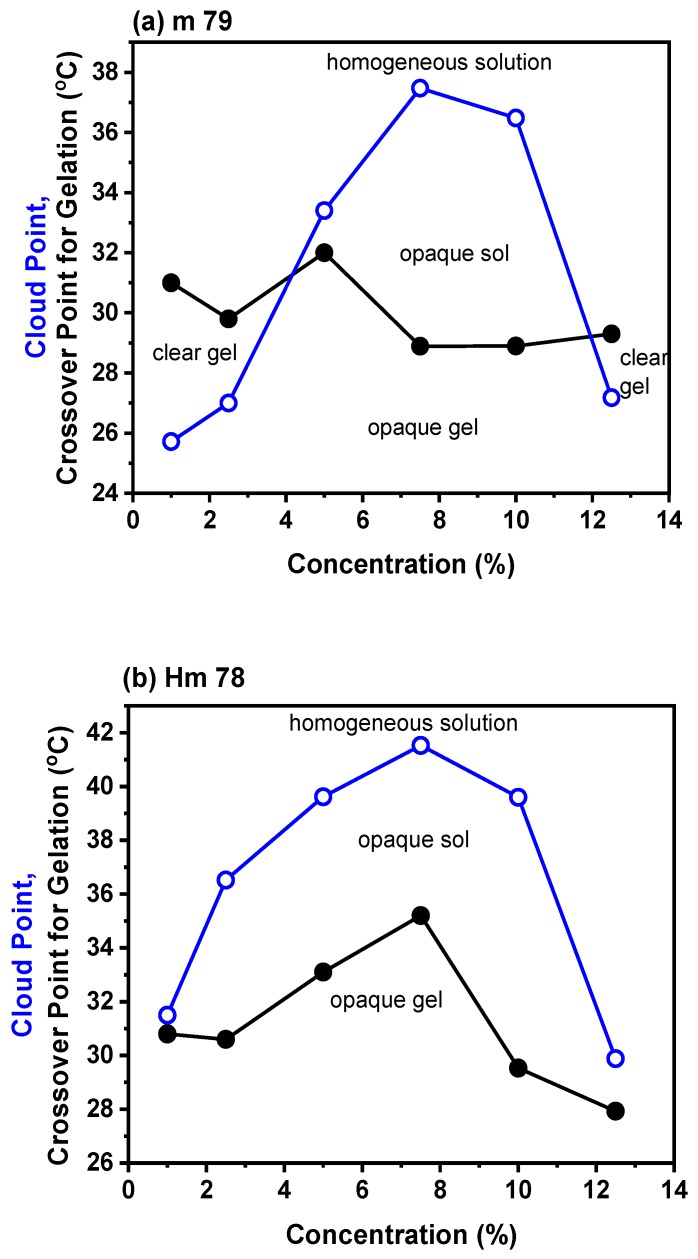
The comparison of the crossover point for gelation (●) and cloud point (○, Reference [24]) for (**a**) m79 and (**b**) Hm78 solutions.

**Scheme 2:**
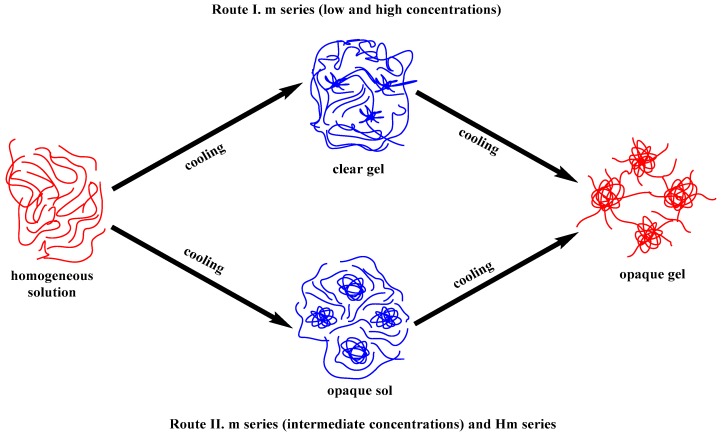
The gelation procedure of m79 and Hm78 in [BMIM][TFSI].

In Figure 10, we compared the crossover points for gelation measured by rheology and the cloud points measured by transmittance test. For m79, the crossover-point line intersects the cloud-point line and phase diagram is divided into four kinds of status. At high enough temperatures that are above both crossover-point and cloud-point boundaries, samples are homogenous solutions. With temperatures decreasing, two different transition routes will happen (illustrated in Scheme 2). At two ends of the phase diagram, samples experience gelation first and then become turbid. The status between gelation and phase separation boundaries is called clear gel, while the status after complete phase separation is called opaque gel. Around the UCST peak, cloud points are higher than the crossover temperature. Therefore, samples experience an opaque sol first before they become an opaque gel. Such interference phenomenon between sol-to-gel and coil-to-globule lines was also reported in isotactic-rich PNIPAM in water [32]. In this system, the gelation phase boundary intersects the cloud-point phase boundary at a concentration of 2% and then drops below at higher concentrations. Their phase diagram was divided into four phases, the same as ours. Tanaka et al. [33] proposed a mechanism to predict such a diagram. In their model, the isotactic-rich PNIPAMs contain two kinds of hydrogen-bonding sites, i.e., isotactic diad and syndiotactic diad, with the latter showing a greater bonding strength with solvents than the former. Therefore, when the temperature is approaching the phase separation boundary, isotactic segments on polymers are desolvated preferentially in comparison to stereo-irregular segments. The desolvated isotactic segments are driven into intermolecular junctions even though other segments are still solvated. By the infrared spectroscopic analysis of the *v* (C–H) region of imidazole ring, Wang et al. [29] experimentally confirmed the desolvation mechanism applies in PNIPAM/IL system, where the interaction experiences a changing process from the dissociation of polymer-ion interaction to the formation of chain-chain bonding, followed by the final chain shrink to aggregation. This mechanism successfully rationalizes the formation of clear gel, which takes place when junctions are formed faster than globules. At concentrations around the CST peak, the formation of globules is faster than the junction, so an opaque sol was formed first across the phase boundary. As gelation further approaches the boundary, the junctions among globules are established so the sol is transformed to an opaque gel.

For Hm78, all crossover points for gelation are located below the cloud points. As a result, only one transition procedure exists, i.e., samples transfer from solutions to opaque sols and then to opaque gels with temperature decreasing. The absence of the clear gel phase could be attributed to the poorer solubility of high-*M*_n_ polymers for entropic reasons, which makes formation of globules as fast as junctions. The crossover-point phase diagram has a similar shape with the cloud-point one. That implies the formation of globules is a dominating procedure for the following gelation.

**Figure 11:**
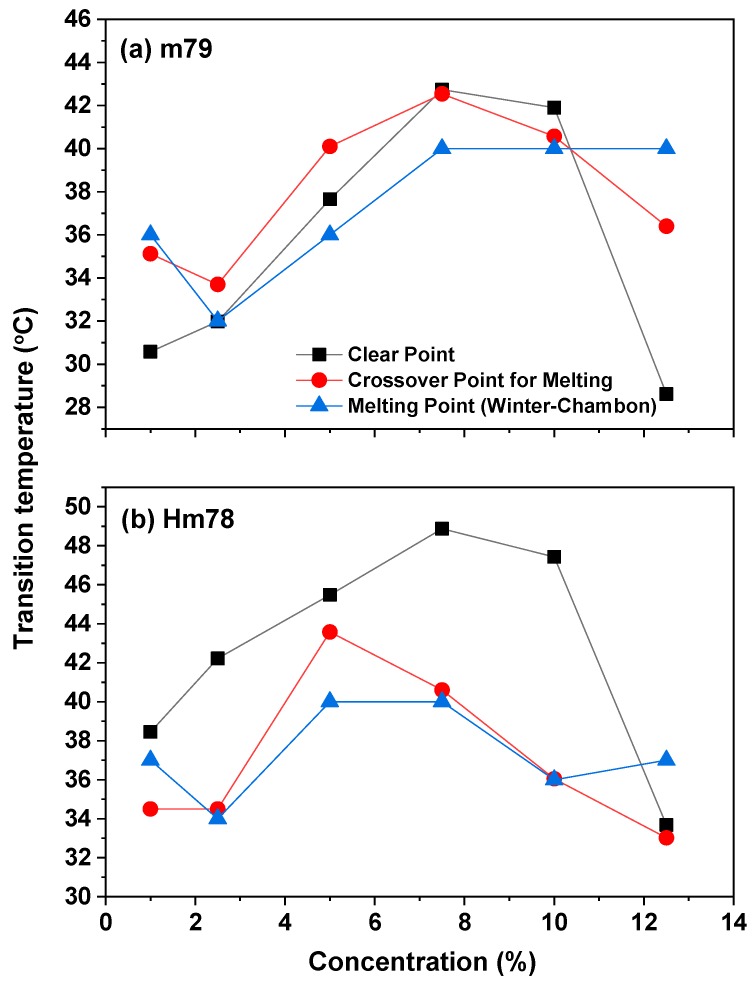
The *G*’-*G*” crossover point for melting (●), the melting point determined by Winter-Chambon criterion (▲), and the clear point determined by transmittance measurement (■), as a function of the concentration for (**a**) m79 and (**b**) Hm78 solutions.

The dynamic frequency sweep at different temperatures was performed on m79 and Hm78 solutions of different concentrations (Figure S5 for m79 and Figure S6 for Hm78 solutions, shown in Supporting Information). The melting temperature based on Winter-Chambon criterion was extracted from the intersecting or closest point of tanδ curves as a function of temperature at different frequencies (Figure S7 for m79 and Figure S8 for Hm78 solutions, shown in Supporting Information). In Figure 11a, the phase diagram of m79 solutions based on Winter-Chambon melting points is established, along with the *G*’-*G*” crossover points for melting and clear points from transmittance measurement. At all investigated concentrations, the difference of transition temperatures between Winter-Chambon and *G*’-*G*” crossover definitions is as small as 4°C, which is within experimental uncertainty. Similar with the case in Figure 10a, both the melting-point line (Winter-Chambon) and the crossover-point line intersect the clear-point line, so the phase diagram is divided into four parts, i.e., homogeneous solution, clear gel, opaque sol, and opaque gel. Such consistency in gelation and melting phase diagrams confirms the validity of the phase transition procedure in Scheme 2. For Hm78 solutions, the phase diagram based on the aforementioned three kinds of transition temperatures is shown in Figure 11b. Again, the difference between Winter-Chambon melting and *G*’-*G*” crossover points is within 4 ℃. All melting points are lower than clear points except the Winter-Chambon value in 12.5% solution, which, however, could be attributed to the experimental uncertainty. Therefore, the validity of gelation phase diagram in Figure 10b is basically confirmed by Figure 11b, in which the phase mainly transfers from homogeneous solutions, to opaque sols, and then to opaque gels, or conversely.

## 4. Conclusions

The sol-gel transition of isotactic-rich PNIPAMs in ionic liquid was investigated by rheological technique. The *G*’-*G*” crossover points for both gelation and melting procedures increase with the meso-diad content, consistent with the coil-globule transition determined by cloud points at the same scanning rate, while the melting point based on Winter-Chambon criterion is of a higher average value (40 ± 2.2 °C) and approximately tacticity and *M*_n_ independent because it corresponds to the percolation point rather than the establishment of elasticity for gels (*G*’ > *G*”). The UCST-type phase diagrams composed of both crossover points for gelation and cloud points are determined. For low-*M*_n_ PNIPAMs (m79), the crossover-point line intersects the cloud-point line, so the phase diagram is divided into four parts: solution, clear gel, opaque sol, and opaque gel. The clear gel is formed because the isotactic segments on the chain experience desolvation preferentially and then act as junctions to connect the solvated segments. When the junction formation is faster than the globule formation, a clear gel appears. Otherwise, an opaque sol will be formed instead of the clear gel prior to the formation of the opaque gel. For high-*M*_n_ PNIPAMs (Hm78), crossover points are below cloud points at all investigated concentrations, so solutions transfer to opaque sol first and then opaque gel with decreasing temperatures. This difference in phase diagram could be attributed to the poorer solubility of high-*M*_n_ polymers for entropic reason. Phase diagrams composed of Winter-Chambon melting points, *G*’-*G*” crossover points for melting, and clear points follow the similar tendency as those based on gelation points, confirming the above transition procedure.

## Figures and Tables

**Table 1:** Characteristics of poly (*N*-isopropylacrylamides) (PNIPAM) Samples.

Sample	*M*_n_ (g/mol)	PDI	Meso-Diad Content (*m*) (%)
m47	35,400	1.25	47
m58	39,500	1.24	58
m66	34,900	1.22	66
m79	40,300	1.26	79
Hm48	62,900	1.49	48
Hm57	60,200	1.26	57
Hm67	62,800	1.32	67
Hm78	85,700	1.38	78

## Supplementary Materials

The following are available online at https://www.mdpi.com/2073-4360/11/5/783/s1, Figure S1: The storage (closed symbol) and loss (open symbol) moduli of m series with concentration of 5% and different tacticity, Figure S2: The storage (closed symbol) and loss (open symbol) moduli of Hm series with concentration of 5% and different tacticity, Figure S3: The loss factor as a function of temperature for m series with concentration of 5% and different tacticity, Figure S4: The loss factor as a function of temperature for Hm series with concentration of 5% and different tacticity. Symbols are the same as in Figure S3, Figure S5: The storage (closed symbol) and loss (open symbol) moduli of m79 solutions with different concentration, Figure S6: The storage (closed symbol) and loss (open symbol) moduli of Hm78 solutions with different concentration, Figure S7: The loss factor as a function of temperature for m79 solutions with different concentration. Symbols are the same as in Figure S3, Figure S8: The loss factor as a function of temperature for Hm78 solutions with different concentration. Symbols are the same as in Figure S3.
