# Supplementary material for: Rheological Study on the Thermoreversible Gelation of Stereo-Controlled Poly(N-Isopropylacrylamide) in an Imidazolium Ionic Liquid
Source: Polymers (Basel). 2019 May 2;11(5):783. doi: 10.3390/polym11050783 (PMC6571980; doi:10.3390/polym11050783)
Supplement: Supplementary file 1 [file polymers-11-00783-s001.pdf]

Supporting Information

Rheological Study on the Thermoreversible Gelation of Stereo-controlled Poly(*N*-isopropylacrylamide) in an Imidazolium Ionic Liquid

Zhi-Chao Yan, [a,*] Chandra Sekhar Biswas,[a] and Florian J. Stadler [a]

[a] College of Materials Science and Engineering, Shenzhen Key Laboratory of Polymer Science and Technology, Guangdong Research Center for Interfacial Engineering of Functional Materials, Nanshan District Key Lab for Biopolymers and Safety Evaluation, Shenzhen University, Shenzhen 518055, People's Republic of China.

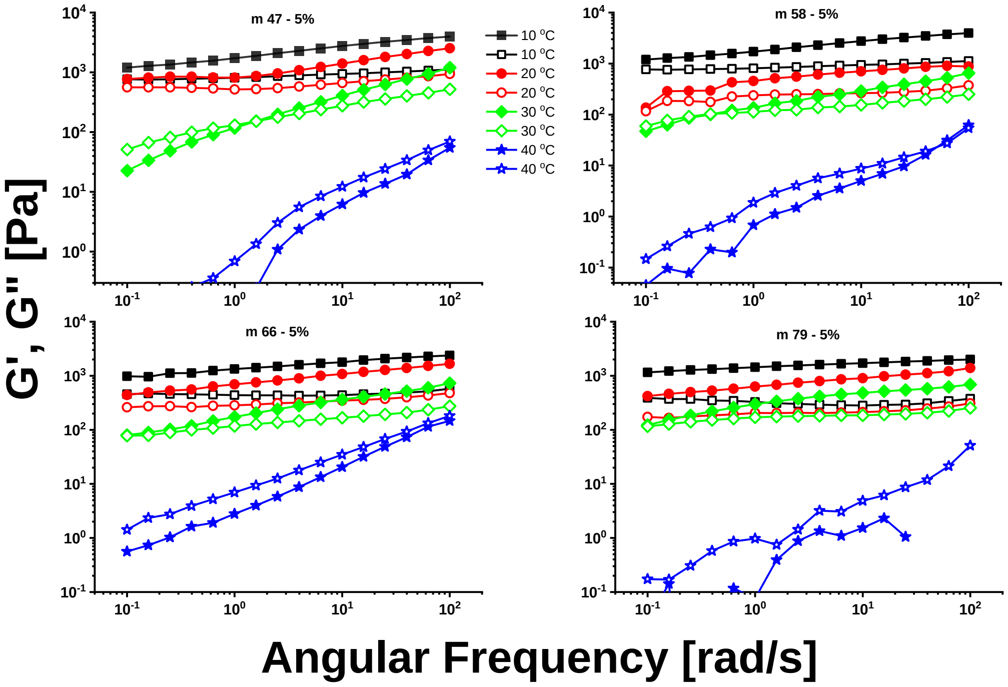

Figure S1. The storage (closed symbol) and loss (open symbol) moduli of m series with concentration of 5% and different tacticity.

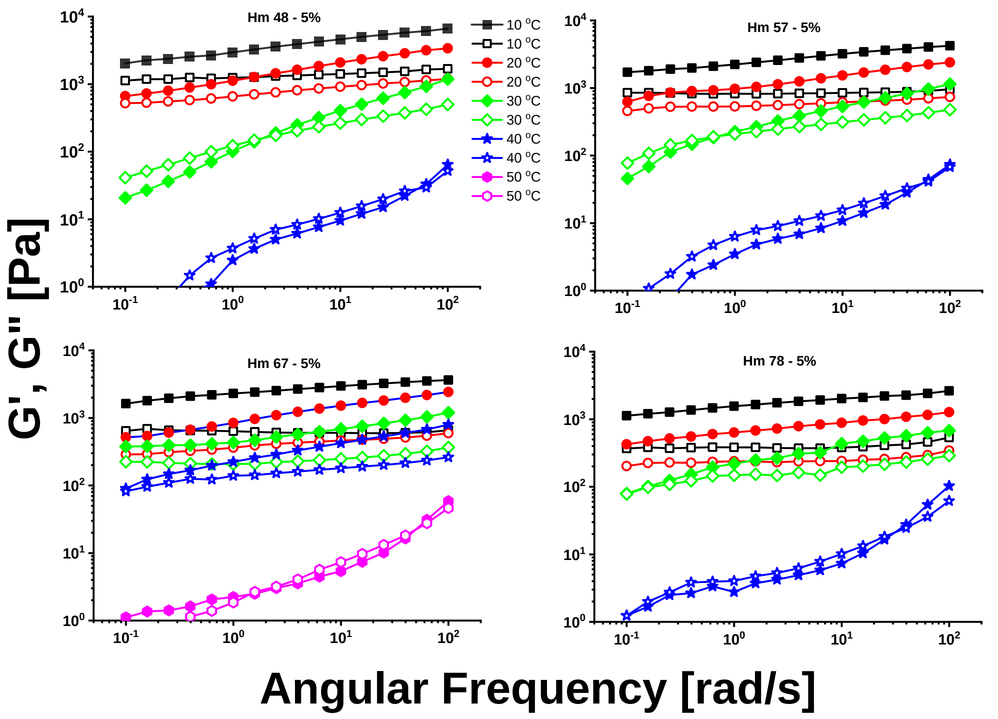

Figure S2. The storage (closed symbol) and loss (open symbol) moduli of Hm series with concentration of 5% and different tacticity.

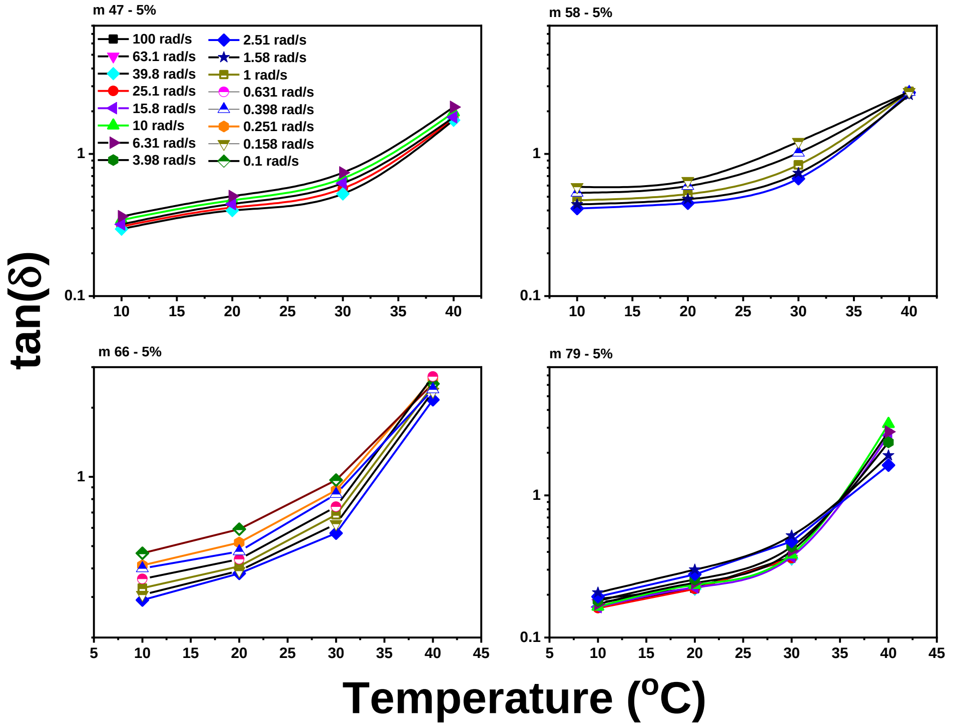

Figure S3. The loss factor as a function of temperature for m series with concentration of 5% and different tacticity.

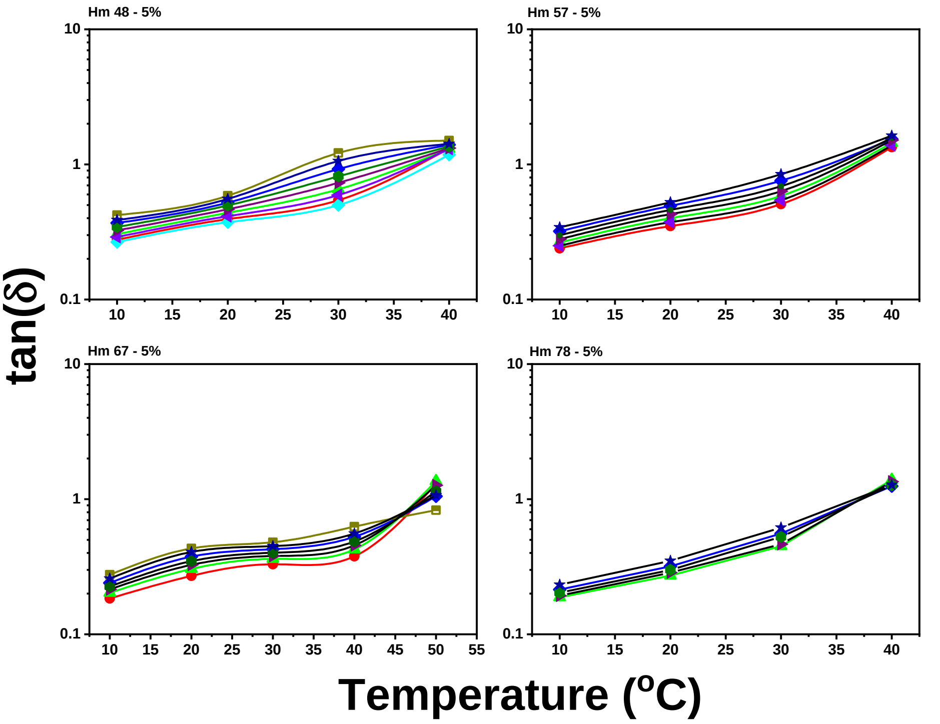

Figure S4. The loss factor as a function of temperature for Hm series with concentration of 5% and different tacticity. Symbols are the same as in Figure S3

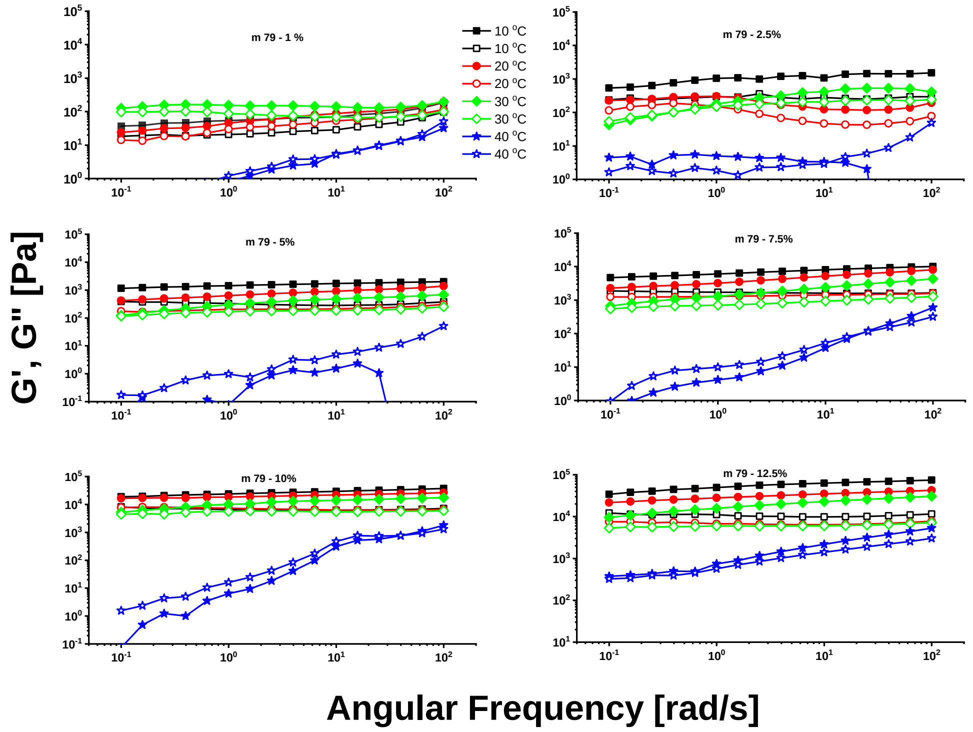

Figure S5. The storage (closed symbol) and loss (open symbol) moduli of m79 solutions with different concentration.

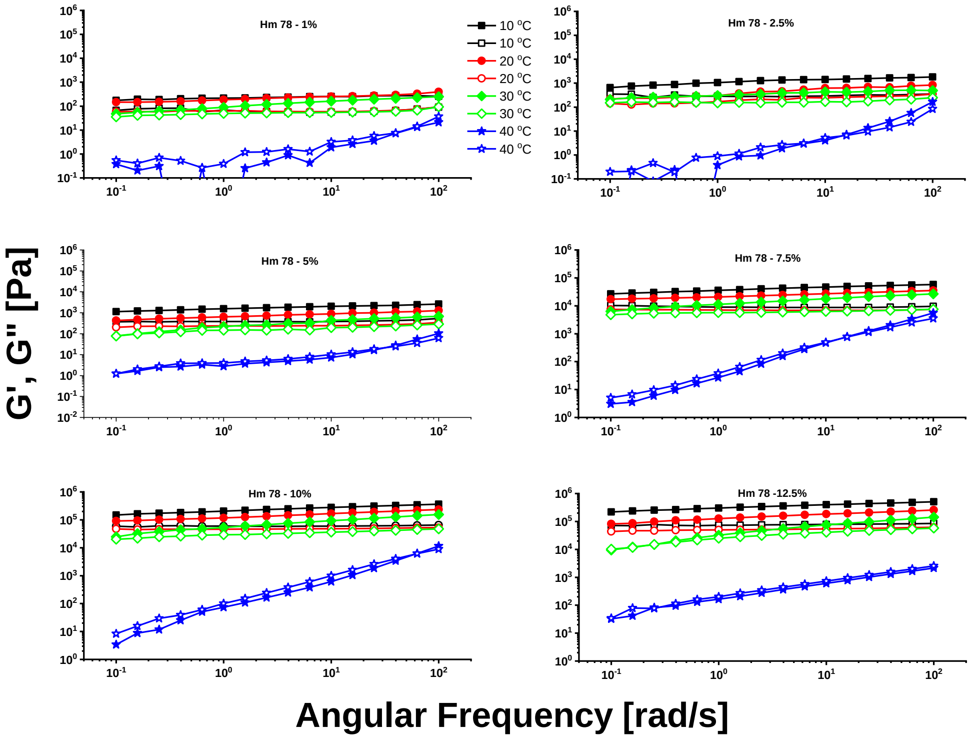

Figure S6. The storage (closed symbol) and loss (open symbol) moduli of Hm78 solutions with different concentration.

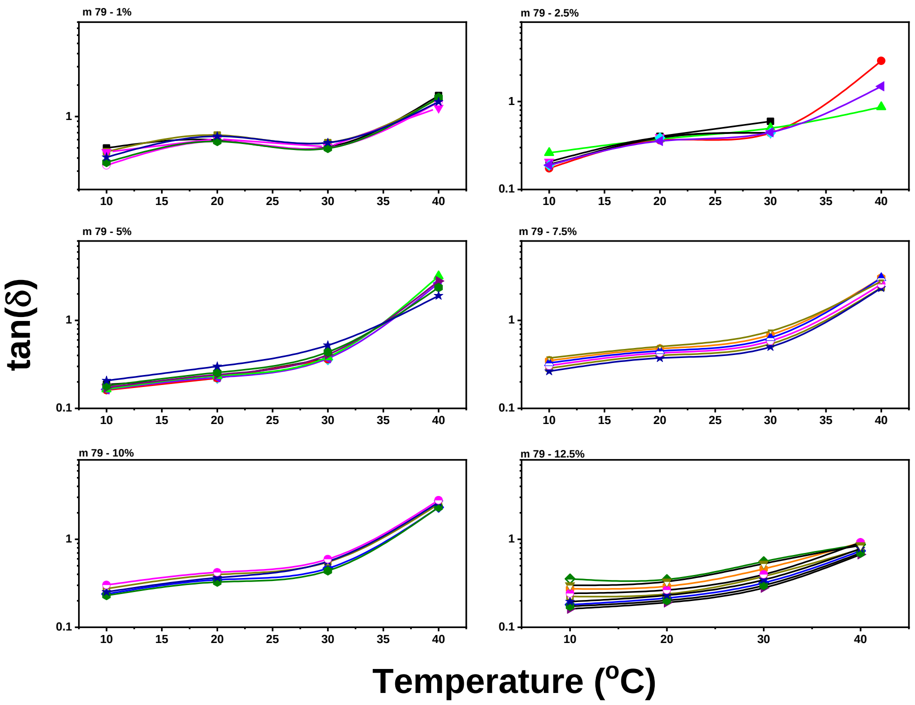

Figure S7. The loss factor as a function of temperature for m79 solutions with different concentration. Symbols are the same as in Figure S3

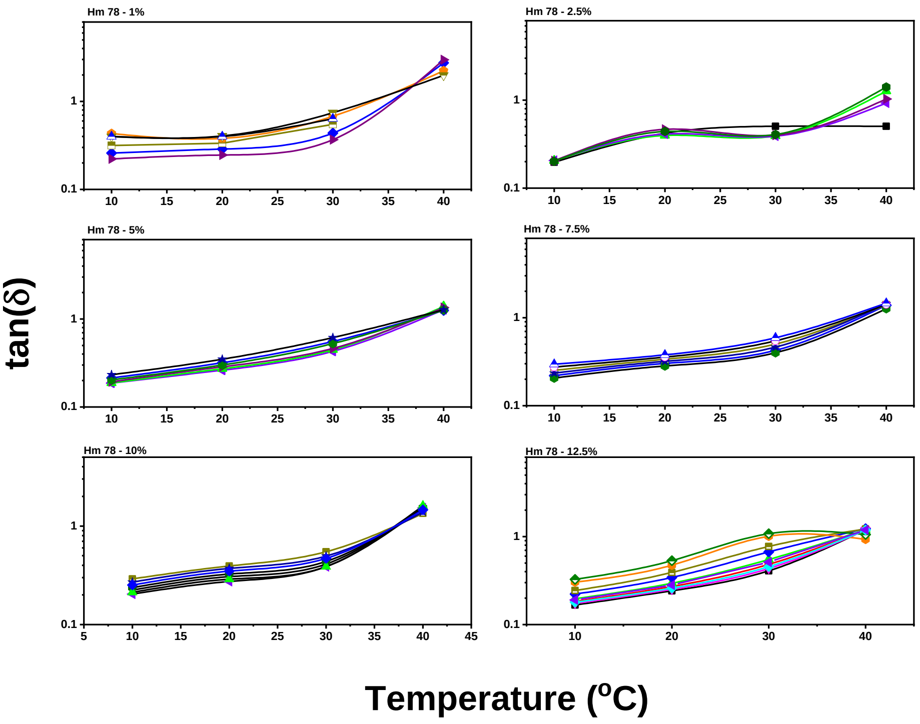

Figure S8. The loss factor as a function of temperature for Hm78 solutions with different concentration. Symbols are the same as in Figure S3
